# Pharmacology, particle deposition and drug administration techniques of intranasal corticosteroids for treating allergic rhinitis

**DOI:** 10.1111/cea.14212

**Published:** 2022-08-23

**Authors:** Corine Rollema, Eric N. van Roon, Job F. M. van Boven, Paul Hagedoorn, Titia Klemmeier, Janwillem H. Kocks, Esther I. Metting, Hanneke N. G. Oude Elberink, Thomas T. A. Peters, Michel R. M. San Giorgi, Tjalling W. de Vries

**Affiliations:** ^1^ Department of Clinical Pharmacy and Pharmacology Medical Centre Leeuwarden Leeuwarden The Netherlands; ^2^ Department PharmacoTherapy, Epidemiology and Economy, Groningen Research Institute of Pharmacy University of Groningen Groningen The Netherlands; ^3^ Department of Clinical Pharmacy and Pharmacology University Medical Centre Groningen Groningen The Netherlands; ^4^ Groningen Research Institute for Asthma and COPD (GRIAC) University Medical Center Groningen, University of Groningen Groningen The Netherlands; ^5^ Department of Pharmaceutical Technology and Biopharmacy, Groningen Research Institute of Pharmacy University of Groningen Groningen The Netherlands; ^6^ Department of Pulmonology Martini Hospital Groningen Groningen The Netherlands; ^7^ General Practitioners Research Institute (GRIP) Groningen The Netherlands; ^8^ Observational and Pragmatic Research Institute Singapore City Singapore; ^9^ Department of Pulmonology University Medical Center Groningen, University of Groningen Groningen The Netherlands; ^10^ Data Science Center in Health University Medical Center Groningen, University of Groningen Groningen The Netherlands; ^11^ Faculty of Economics and Business University of Groningen Groningen The Netherlands; ^12^ Department of Allergology University Medical Center Groningen, University of Groningen Groningen The Netherlands; ^13^ Department of Otorhinolaryngology Medical Centre Leeuwarden Leeuwarden The Netherlands; ^14^ Department of Otorhinolaryngology, Head and Neck Surgery University Medical Center Groningen Groningen The Netherlands; ^15^ Department of Paediatrics Medical Centre Leeuwarden Leeuwarden The Netherlands

## Abstract

This review presents an overview of the available literature regarding intranasal corticosteroids (INCs) for the treatment of allergic rhinitis (AR). Various treatment options exist for AR including INCs, antihistamines and leucotriene antagonists. INCs are considered to be the most effective therapy for moderate‐to‐severe AR, as they are effective against nasal and ocular symptoms and improve quality of life. Their safety has been widely observed. INCs are effective and safe for short‐term use. Local adverse events are observed but generally well‐tolerated. The occurrence of (serious) systemic adverse events is unlikely but cannot be ruled out. There is a lack of long‐term safety data. INC may cause serious eye complications. The risk of INCs on the hypothalamic–pituitary–adrenal axis, on bone mineral density reduction or osteoporosis and on growth in children, should be considered during treatment. Pharmacological characteristics of INCs (e.g. the mode of action and pharmacokinetics) are well known and described. We sought to gain insight into whether specific properties affect the efficacy and safety of INCs, including nasal particle deposition, which the administration technique affects. However, advances are lacking regarding the improved understanding of the effect of particle deposition on efficacy and safety and the effect of the administration technique. This review emphasizes the gaps in knowledge regarding this subject. Advances in research and health care are necessary to improve care for patients with AR.

**Key points**

- INCs are effective and safe for short‐term use, but rarely cause serious eye complications.
- INCs' efficacy and safety may depend on nasal particle deposition, which the administration technique affects.
- It is not clear how each step of the administration technique affects particle deposition.

## 1 INTRODUCTION

Intranasal corticosteroids (INCs) are the cornerstone treatment when persistent symptoms of allergic rhinitis (AR) occur. AR has a significant negative impact on patients' quality of life (QoL) and adds to healthcare costs. Advances in the treatment with INCs are lacking, especially in regard to the improved understanding of particle deposition and administration techniques, in contrast to the scientific understanding of these topics for the treatment of lung diseases such as asthma.^1^ We focus on INCs because they are the cornerstone pharmacological treatment option for moderate‐to‐severe AR. In this review, we attempt to combine the knowledge about the efficacy and safety and the influence of specific properties of INCs, including particle deposition and the effect of the administration technique. Based on this, we will identify gaps in knowledge and provide recommendations for future research.

## 2 SEARCH METHODOLOGY

To identify relevant literature to inform this narrative review, we performed a semi‐structured search in PubMed with combinations of the following search terms: allergic rhinitis, internasal corticosteroids, pharmacology, deposition and administration technique. Full texts of manuscripts deemed relevant were inspected, and additional papers were retrieved by searching the references.

## 3 ALLERGIC RHINITIS AND TREATMENT OPTIONS

Allergic rhinitis is a global health problem in children and adults. Its prevalence ranges from 8.5% to 27.2%, depending on age and geography.^2^, ^3^ AR may lead to QoL impairments and sleep problems.^4^, ^5^, ^6^ In children, this may result in decreased academic performances.^5^ In adults, it reduces work productivity.^7^ The economic burden of AR is substantial, with overall costs for medical treatment an estimated $7.3 billion and work productivity losses an estimated $4.3 billion in the United States in 2002.^4^

**FIGURE 1:**
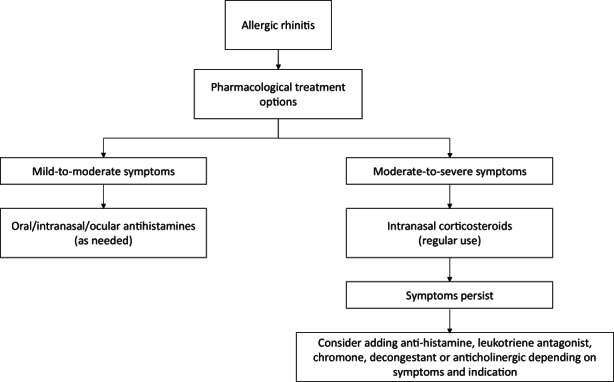
Flowchart for the treatment of allergic rhinitis based on Table 1.^4^, ^5^, ^104^, ^105^

Pharmacological treatment options available for AR include INCs, antihistamines, leucotriene antagonists, decongestants, anticholinergics, chromones, saline rinses and immunotherapy, which are mainly administered intranasally or orally.^5^, ^7^ Selection of the optimal treatment approach depends on the temporal pattern, frequency and severity of symptoms (i.e. mild and moderate‐to‐severe).^7^ Table 1 and Figure 1 provide an overview of pharmacological options for the treatment of AR, including their characteristics and how to apply treatment options.

**TABLE 1:** Overview of pharmacological treatment options for the treatment of AR.^4^, ^5^, ^7^, ^25^, ^46^, ^104^, ^105^

Treatment option	Route of administration	Including	Effective against	Less effective against	Effect within	Effect lasting	Sufficient for	Position in practice guidelines
Corticosteroids	Intranasal	Beclomethasone dipropionate Budesonide Ciclesonide Fluticasone furoate Fluticasone propionate Mometasone furoate Flunisolide Triamcinolone acetonide	Overall AR symptoms Ocular symptoms Nasal congestion		12 h	24 h	Moderate‐to‐severe symptoms	ARIA: **1st choice** for seasonal and persistent AR AAO‐HNSF: For patients with a clinical diagnosis of AR whose symptoms affect their quality of life
	Oral	Predniso(lo)ne			—	—	In a short period (5–7 days) for very severe nasal symptoms	ARIA: A short course for moderate to severe nasal and/or ocular symptoms that are not controlled with other treatments
Antihistamines	Oral (2nd generation)	Acrivastine Bilastine Cetirizine Desloratadine Ebastine Fexofenadine Levocetirizine Loratadine Mizolastine Rupatadine	Rhinorrhoea Sneezing Nasal itching Ocular symptoms	Nasal congestion	1–2 h	12–24 h	Mild‐to‐moderate symptoms	ARIA: **2nd choice** for seasonal and persistent AR AAO‐HNSF: For patients with AR and primary complaints of sneezing and itching
	Intranasal	Azelastine Levocabastine	Nasal congestion Nasal itching Sneezing Runny nose		1–2 h	12–24 h	Mild‐to‐moderate symptoms	ARIA: **3rd choice** for seasonal AR AAO‐HNSF: For seasonal, perennial, or episodic AR
	Ocular	Azelastine Emedastine Ketotifen Levocabastine Olopatadine	Allergic eye symptoms (conjunctivitis)		3–10 min, sometimes 30 min	4–8‐12 h	Eye symptoms	ARIA: For seasonal and persistent AR with conjunctivitis
Leucotriene antagonist	Oral	Montelukast	Nasal symptoms (alone or in combination with antihistamines)		2 h	—	Not as primary therapy used in patient with both AR and asthma	ARIA: **4th choice** for seasonal AR AAO‐HNSF: Not offered as primary therapy for patients with AR
Chromones	Intranasal	Cromolyn sodium	Nasal itching Rhinorrhoea Sneezing	Nasal congestion	Couple of days, sometimes weeks	3–4 h	Adjunct therapy Mild symptoms	ARIA: **5th choice** for seasonal and persistent AR
	Ocular	Cromolyn sodium	Allergic eye symptoms (conjunctivitis)		Couple of days	3–4 h	Eye symptoms	ARIA: For seasonal and persistent AR with conjunctivitis
Decongestants/imidazoline derivates	Intranasal	Oxymetazoline Tramazoline Xylometazoline	Nasal congestion		5 min	8–12 h	Adjunct therapy Mild symptoms	ARIA: A very short course for seasonal and persistent AR and severe nasal obstruction with other treatment
	Oral	Pseudoephedrine Phenylephrine	Nasal congestion		—	—		ARIA: Not suggested as therapy regularly
Anticholinergics	Intranasal	Ipratropium	Rhinorrhoea	Nasal symptoms	15 min	4–6 h	Adjunct therapy Mild symptoms	ARIA: For persistent AR with rhinorrhoea

*Note*: AAO‐HNSF: American Academy of Otolaryngology—Head and Neck Surgery. Clinical Practice Guideline:Allergic Rhinitis.^104^ ARIA: Allergic Rhinitis and its Impact on Asthma. Guidelines: 2010 Revision.^105^

## 4 EFFICACY AND SAFETY OF INTRANASAL CORTICOSTEROIDS

In this review, we will discuss aspects regarding treatment with INCs in depth. Several devices are available for nasal administration of INCs (Table 2). The efficacy and safety of INCs have been analysed extensively in the literature.

**TABLE 2:** Overview of the devices that are available for intranasal administration of corticosteroids.^9^, ^18^, ^43^, ^106^, ^107^, ^108^, ^109^, ^110^, ^111^

Device	INC	Brand name	Generic	FDA	EMA	Comments
Metered‐dose spray pump	Azelastine/fluticasone propionate	Dymista®		✓	✓	Available as an aqueous formulation in a spray pump. After activation of the spray tip, a spray plume is created. Popular for its ease of use. In 1987, the first INC spray was approved by the FDA
	Beclomethasone dipropionate	Alanase® Beconase® Rivanase®	✓	✓	✓	
	Budesonide	Rhinocort®	✓	✓	✓	
	Fluticasone furoate	Avamys® Veramyst®		✓	✓	
	Fluticasone propionate	Flonase® Flixonase® Ticanase®	✓	✓	✓	
	Mometasone furoate	Nasonex®	✓	✓	✓	
	Triamcinolone acetonide	Allernaze® Nasacort®	✓	✓	✓	
Pressurized metered‐dose inhaler (pMDI)	Beclomethasone diproprionate	Qnasl®		✓		In 1980–1990 the first INC pMDI came on the market with chlorofluorocarbon (CFC) as propellant. Due to disadvantages for the patient and for the ozone layer all CFC‐propellant products were removed from the market in 2003. In 2012, hydrofluoroalkane (HFA) as propellant was approved by the FDA
Dry powder device	Budesonide	Rhinocort® Turbuhaler®		Discontinued	Discontinued	A breath‐actuated dry powder formulation. Does not contain any propellants, preservatives or additives. An aerosol is created when the powder particles are inhaled through the nose
Nasal drops	Fluticasone propionate	Flixonase®			✓	The solvent including INC is dropped into the nose by a dropper or directly from an ampoule. When the drops are administered correctly, penetration occurs beyond the nasal valve, which is beneficial for nasal polyps
Exhalation delivery system (EDS)	Fluticasone propionate	Xhance®		✓		Approved for nasal polyps. When the patient blows through the mouthpiece of the EDS, the device is activated. The mouthpiece is connected to an INC reservoir and via the nosepiece the medication flows into one nostril. The working substance reaches deeper into the nose (superior/posterior regions), which causes higher efficacy and leads to less drip‐out and swallowing
Nebulizing solution						Based on use in daily clinical practice. Contains INC drops and/or a saline solution. Can be used for severe nasal polyps. This nebulizer solution reaches deeper into the nose and is therefore more effective for severe indications
Nasal rinse						Based on use in daily clinical practice. Can be used as a tool to administer solutions in clinical practice. Mainly containing a saline solution to rinse the nose

Abbreviations: EMA, European Medicines Agency; FDA, United States Food & Drug Administration; INCs, intranasal corticosteroid.

### 4.1 Efficacy

Analysing the efficacy of INCs uses both subjective (i.e. patient‐reported) and objective outcome measures. Moreover, a distinction is made between clinical trial data and real‐world data.

#### Randomized clinical trial data

Randomized clinical trial (RCT) and meta‐analysis data indicate that regular use of INCs is most effective for moderate‐to‐severe AR symptoms compared with other treatment options.^5^ Table 1 and Figure 1 provide an overview of the different treatment options and their efficacy. Meta‐analysis concludes that INCs are effective against nasal and ocular symptoms and improve QoL.^5^, ^8^ No clear evidence conveys that one INC is more effective than another.^9^, ^10^ For mild‐to‐moderate AR symptoms, INC therapy is not recommended, although studies have suggested that INCs may be effective as as‐needed therapy for mild AR symptoms, compared with placebo and antihistamines.^11^, ^12^, ^13^ Hoang et al.^13^ emphasize that regular use of INCs provides greater benefits than the as‐needed therapy in total nasal symptoms score and disease‐specific QoL.

#### Emerging efficacy data: Nasal obstruction

Beyond the classic outcomes such as nasal and ocular symptoms, objective parameters that measure nasal obstruction are the peak nasal inspiratory flow (PNIF), acoustic rhinometry and rhinomanometry.^5^, ^14^ Interest in using PNIF in daily practice is increasing, because it is simple, inexpensive, fast and reproducible.^5^, ^14^ Studies have shown significant improvements of the PNIF in adults in INC‐treatment groups compared with placebo groups.^15^, ^16^

There appears to be a discrepancy between subjective determination of nasal obstruction and the aforementioned objective scores.^14^, ^17^ The question remains as to whether these objective parameters are of added value in clinical practice, considering that a patient's experience of nasal airflow and obstruction remains primarily a subjective parameter.^17^ Furthermore, the effect of an improved nasal passage on patients' experience of disease impact is questionable. The Bernoulli effect refers to when a fluid (i.e. liquid or gas) flows through a tube of varying diameters (e.g. the nose) and passes through a narrowing (e.g. the nasal valve), causing the local speed of the fluid to increase and its pressure to decrease. This causes suctioning of the nasal valve, leading to a smaller valve and resulting in further pressure decrease and occasionally a total blockage of the nasal airway during inspiration.^18^ An improved nasal passage therefore does not necessarily lead to better airflow and should not necessarily be the aim of pharmacological treatment.

#### Data from real‐world daily practice

While RCTs provide efficacy data from mostly high‐controlled environments, INC effectiveness in real‐world daily practice may differ due to more heterogeneous populations and less strict monitoring procedures. The number of studies on real‐world effectiveness is limited, as are details on efficacy according to age or AR phenotype. Scadding et al.^19^ analysed the efficacy of INCs using a self‐developed questionnaire and found that on major AR symptoms and adverse events, the profile of INCs is similar in clinical practice and RCTs. Bukstein et al.^20^ assessed the effectiveness of a nonaqueous beclomethasone dipropionate nasal aerosol using multiple validated patient‐reported outcome measures (i.e. AR symptoms, QoL, work and school performances and sleep quality). The overall efficacy of INCs has been confirmed.^20^ However, another study mentions that patients in daily practice seek treatment when symptoms occur and stop treatment when symptoms are under control.^21^ It is likely that this negatively affects medication adherence. Indeed, multiple studies have concluded that adherence to the INCs therapy must improve in children and adults.^22^, ^23^

### 4.2 Safety

Research into the safety of INCs is widely available. A clear distinction is made between local and systemic adverse events.

#### Local adverse events

Local irritation and dryness of the nose and throat and sneezing after administration are common local side effects of INCs.^24^, ^25^ All INC sprays are associated with a significantly increased risk of epistaxis compared with placebo or no intervention, according to two reviews with relative risk 1.48 (95% CI: 1.32–1.67, 72 studies) and risk ratio 2.74 (95% CI: 1.88–4.00, 2508 participants, 13 studies), respectively.^26^, ^27^ In the Cochrane review, the number needed to harm (NNH) can be extracted (NNH = 20 [95% CI: 12–49]).^27^ The cause of epistaxis is not entirely clear. One possible explanation is that the majority of the INCs dose impinges on the anterior septum, which contains the highest density of blood vessels (Kiesselbach's plexus) and thin mucosa, which makes this part of the nose vulnerable.^28^ Another explanation is the chemical and direct trauma caused by the corticosteroid and its spray tip.^29^ Serious local adverse events such as atrophy of the nasal mucosa or septal perforation are rare.^30^ Overall, INCs are generally well tolerated, and the number of side effects reported—which are generally mild—is limited.^8^

#### Systemic adverse events

As a result of the low systemic bioavailability of INCs, the risk of developing systemic adverse events is likely to be relatively low. Research has been conducted on the occurrence of systemic adverse events, because they occur frequently when using oral or inhaled corticosteroids.^30^ Studies have suggested that INCs do not significantly affect the hypothalamic–pituitary–adrenal axis in children (i.e. aged 3 or older) or adults.^31^, ^32^, ^33^, ^34^ However, several RCTs have studied the effects of INCs on the growth of children (i.e. aged 3 or older) and demonstrated contrasting results.^32^, ^35^, ^36^, ^37^ In some studies, no significant changes were found, but a temporary reduction in short‐time growth velocity was evident.^32^ No evidence currently demonstrates that INCs are associated with bone mineral density reduction or osteoporosis in children (i.e. aged 6 years or older) or adults.^30^, ^38^ A meta‐analysis concludes that based on their results, INCs are not associated with a significant increased risk of elevating the intraocular pressure (relative risk 2.24 [95% CI: 0.68–7.34, 494 studies]) or developing cataracts (absolute increased incidence 0.02% [95% CI: −0.3% to 0.4%, 494 studies]). An association with the risk of occurrence of glaucoma cannot be ruled out.^13^ The association between INCs and the occurrence of chorioretinopathy is rare.^39^ A limitation of most of these studies is the short study period (maximum 1 year). Follow‐up research is therefore still necessary.

## 5 NASAL PARTICLE DEPOSITION

Intranasal corticosteroid efficacy and safety may depend on nasal particle deposition. However, this is given scant attention in the scientific literature. We discuss the current knowledge in more detail in the sections below. “Particles” or “Droplets” refer to particles and droplets with an aerodynamic diameter, which are corrected for dynamic shape factor and density.

### 5.1 Anatomy of the nose

The anterior opening of the nostril is called the vestibule, which leads into a narrow triangular‐shaped slit, called the valve.^18^, ^40^ This anterior nasal cavity is covered with non‐ciliated epithelium containing hair and extends into the posterior nasal cavity.^18^ The posterior nasal cavity has a larger surface area than the anterior nasal cavity and is covered with ciliated epithelium with respiratory mucus, which is secreted by goblet cells.^18^, ^40^ The epithelial cells in the posterior nasal cavity contain microvilli that increase the contact surface area and are important for transport (i.e. mucociliary flow).^18^ Certain factors may influence the mucociliary flow (e.g. drugs that contain the preservative benzalkonium chloride reduce or irreversibly inhibit the mucociliary flow).^41^, ^42^ Three nasal turbinates (i.e. superior, middle and inferior) divide the posterior nasal cavity into narrow passages.^18^, ^43^ The space between the turbinates and nasal wall is called a meatus.^23^ Sinus openings are located in the middle and superior meatus.^40^, ^43^ Sinus outflow may cause congestion and swelling.^40^ The posterior part of the nose merges into the nasopharynx, where the adenoids are located.^44^ Further drainage occurs through the throat via the pharynx and larynx.

### 5.2 Mode of action

**FIGURE 2:**
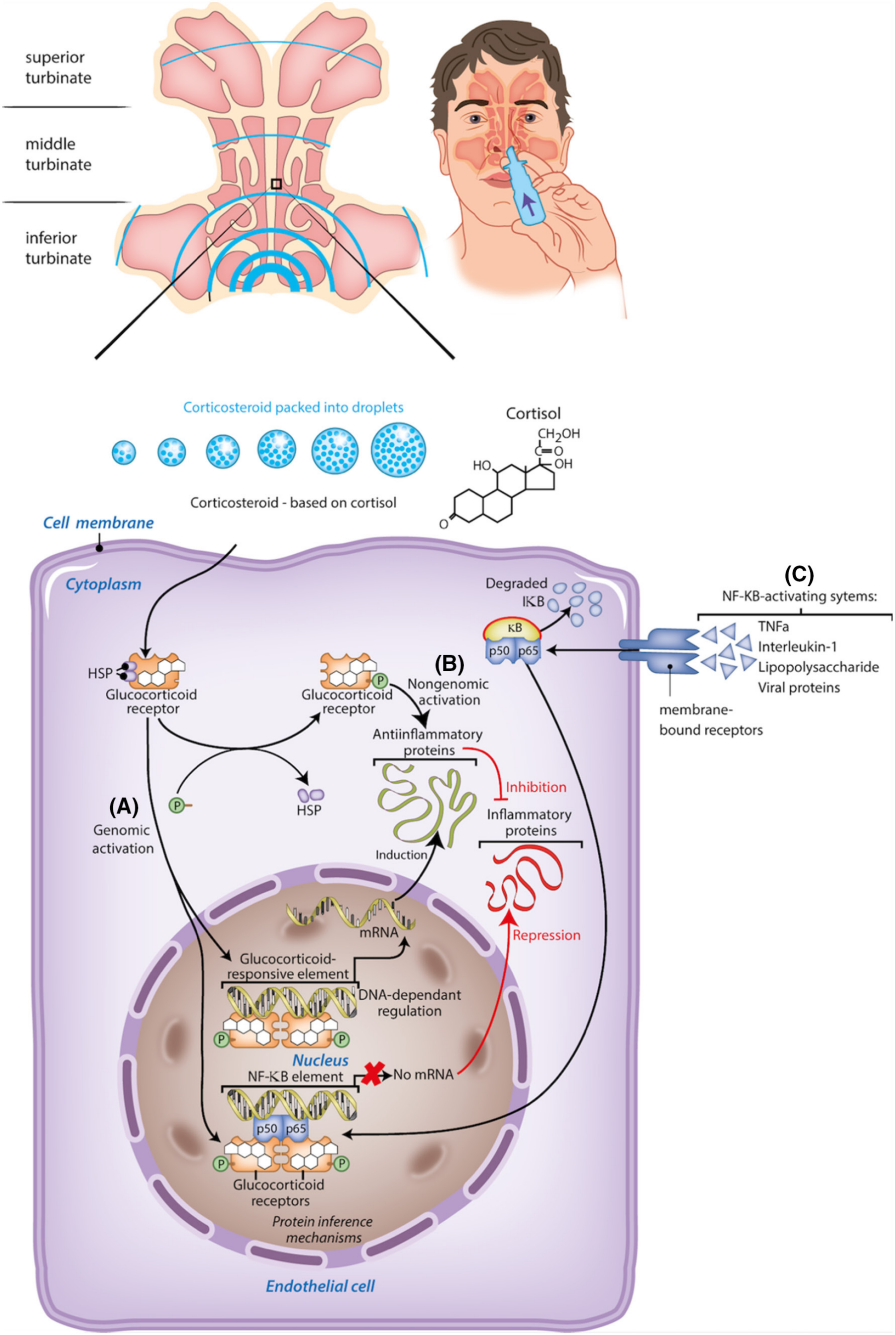
Mode of action of glucorticoids (GCs) —three mechanisms. (A) After diffusion across the cell membrane, GCs bind to the glucocorticoid receptor (GR). The GC/GR complex is translocated to the nucleus and binds to the DNA GC/GR complex (genomic activation). This increases the transcription of genes that encode for anti‐inflammatory proteins (transactivation) and suppresses the transcription of genes that encode for pro‐inflammatory and immune proteins (transrepression). (B) By dissociation of the GC/GR complex, GC signalling through membrane‐associated receptors and second messengers is activated (nongenomic activation). (C) The GC/GR complex interacts with other transcription factors, such as nuclear factor‐κB. This prevents the production of inflammatory proteins.

Glucocorticoids (GCs) suppress many stages of the allergic inflammation.^5^ Figure 2 presents an overview of the mode of action (MOA). In reaction to allergic stimuli, GCs block the synthesis and release of inflammatory mediators and thereby reduce the influx of inflammatory cells into the nasal mucosa.^45^ Several mechanisms of action are involved. The primary mechanism is that after diffusion across the cell membrane, GCs bind to the glucocorticoid receptor (GR).^46^, ^47^ The GC/GR complex is translocated to the nucleus and binds to the DNA GC/GR complex (i.e. genomic activation).^47^ This increases the transcription of genes that encode for anti‐inflammatory proteins (i.e. transactivation) and suppresses the transcription of genes that encode for pro‐inflammatory and immune proteins (i.e. transrepression).^46^ Second, the GC/GR complex interacts with other transcription factors, such as nuclear factor‐κB, which prevents the production of inflammatory proteins.^46^, ^48^ Third, by dissociation of the GC/GR complex, GC signalling through membrane‐associated receptors and second messengers is activated (i.e. nongenomic activation).^46^, ^48^

### 5.3 Pharmacokinetics

Intranasal corticosteroids are characterized by differences in the affinity for the GR, topical potency, systemic bioavailability, rate of hepatic clearance and systemic elimination.^47^ The available INCs compounds differ in these pharmacokinetic characteristics, mainly due to their absorption properties, including lipid solubility.^38^ Increased lipophilicity correlates with a higher and faster uptake by the nasal mucosa, leading to greater retention within the nasal tissue, more time to bind to the GR in the tissue and consequently less unbound fraction, which potentially interacts with systemic GRs and results in adverse events.^30^, ^49^, ^50^, ^51^ The ranked order of INCs, from highest to lowest lipid solubility, is mometasone furoate, fluticasone propionate, beclomethasone dipropionate, budesonide, triamcinolone acetonide and flunisolide.^38^, ^50^ Notably, the effect of these differences in lipid solubility on clinical outcomes remains unclear, but lipid solubility has been shown to be highly correlated with GR affinity.^38^, ^51^ GR binding affinity correlates with the therapeutic dose. Newer INCs molecules (e.g. fluticasone furoate, fluticasone propionate and mometasone furoate) have a higher GR binding affinity compared with older INCs molecules (e.g. beclomethasone dipropionate, budesonide and dexamethasone) and the therapeutic daily dose is therefore lower.^51^ However, GR binding affinity is not the key factor driving topical potency. Topical potency also depends on the deposition pattern and uptake and retention in nasal tissue. There seems to be a relationship between the pharmacokinetic properties and clinical efficacy; higher GR binding affinity and topical potency can potentially improve the therapeutic index (i.e. measurable systemic activity divided by the therapeutic dose) of an INCS.^51^

#### Systemic bioavailability

The systemic bioavailability of INCs is primarily determined by the minimal fraction that is absorbed in the nasal mucosa. Secondly, the largest amount of INCs is swallowed, absorbed by the gastro‐intestinal tract and cleared by the first‐pass metabolism.^38^ Commonly used INCs (e.g. mometasone furoate, fluticasone propionate, fluticasone furoate and ciclesonide) have pharmacokinetic properties that minimize systemic bioavailability (<1%) compared with other INCs (e.g. triamcinolone acetonide, flunisolide, beclomethasone and dexamethasone) and oral corticosteroids.^30^, ^49^, ^50^

### 5.4 Desired deposition pattern

When analysing the deposition pattern of INCs, it is important to determine where INCs droplets must be deposited. Benninger et al.^40^ state that a high deposition of INCs is required in the middle and inferior turbinates and in the middle meatus where sinus outflow congestion and swelling occur. Moreover, they suggest targeting ciliated cells in the mucosa on the lateral wall, in order to distribute the product more widely.^40^ Vidgren and Kublik^52^ note that a wide distribution on the mucosa is required for local efficacy. Blaiss et al.^49^ and Homer et al.^53^ state that INCs should pass beyond the nasal valve with significant deposition in the middle meatus and minimal deposition in the pharynx. Weber et al.^54^ mention that particles that remain on the anterior portion of the nasal septum and the head of the inferior turbinate lead to effective AR control. The optimal deposition pattern therefore remains unclear.

### 5.5 Identified deposition patterns

Different in vitro studies have been conducted to analyse where INCs particles are actually deposited. Benninger et al.^40^ found that particles are mainly deposited in the inferior and middle turbinates. Djupesland et al.^43^ state that deposition is mainly influenced by the spray plume (i.e. diameter ± 2 cm) that the device creates. As a result, the majority of the particles impinge on the non‐ciliated mucosal walls of the vestibule and the narrow valve. Particles that do pass the valve are mainly from the lower and wider part of the triangular plume.^43^

In vivo studies analysing the deposition pattern of INCs are limited. Weber et al.^54^ analysed 35 nasal cavities of 18 patients with video endoscopy. They demonstrated that most particles administered with an INC spray are deposited in the anterior non‐ciliated part of the nose and the head of the inferior turbinate. A small fraction reaches the middle turbinate.^54^ Homer et al.^53^ studied 10 nasal cavities using a radio‐labelled aqueous spray and found a wide variation in the quantity of absorbed administered substance in the middle meatus.^53^ Senocak et al.^55^ used computed tomography to study 14 nasal cavities. Particles were detected in the middle meatus in one case, in the middle turbinate in two cases and in the inferior turbinate in seven cases.^55^

### 5.6 Administration technique of intranasal corticosteroids

The deposition pattern of INCs in the nose may be influenced by the administration technique. Because most INC products are available in spray pumps and because these delivery devices are most commonly used, we focus on the particle deposition of INC sprays. INCs particles in INC sprays are packed in droplets.

Only one review focused on evaluating the correct administration technique of INCs. Benninger et al.^40^ found no clear relationship between the INC administration technique and the effect on efficacy and safety. They recommended a standard administration technique.^40^ It is remarkable that in a random selection of 25 RCTs that analysed the efficacy of INCs, only five RCTs mentioned the administration technique in the methodology section. One to three instruction steps were mentioned (e.g. holding breath before, during or after administration; closing the nostril; and sniffing after administration).^56^, ^57^, ^58^, ^59^, ^60^, ^61^, ^62^, ^63^, ^64^, ^65^, ^66^, ^67^, ^68^, ^69^, ^70^, ^71^, ^72^, ^73^, ^74^, ^75^, ^76^, ^77^, ^78^, ^79^, ^80^

**FIGURE 3:**
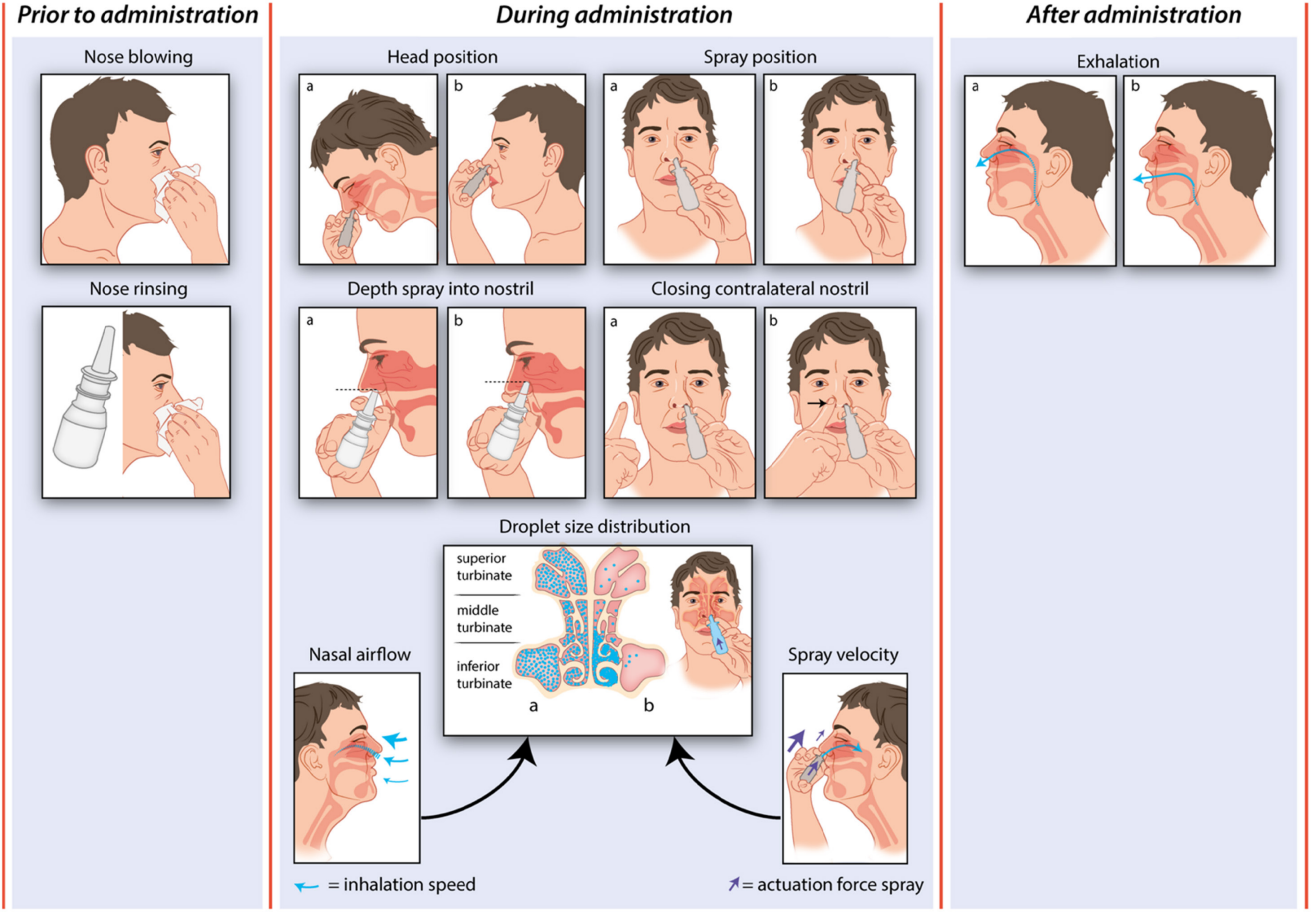
Administration technique and deposition pattern. In the figure, the possible variations in the instruction steps prior to, during and after administration of an INC spray are displayed, which affect the deposition pattern of INC particles. The instructions steps include instructions regarding blowing and rinsing the nose prior to administration, instructions regarding the head position, the spray position, the depth of the spray into the nostril, closing the contralateral nostril and the droplet‐size distribution determined by the nasal airflow and the spray velocity during administration and the instruction regarding exhalation after administration. As described in: Section 5.4, from this review, no definitive conclusions regarding which administration technique leads to the desired deposition pattern and highest efficacy can be drawn for all instruction steps. To give insight in how an instruction step can be carried out differently, the figure illustrates the possible variation in these instruction steps. If a variation in the instruction step is possible, two examples of the variation are displayed as illustration a and b. In this figure, no recommendations regarding the correct administration technique according the authors are indicated. For this, we refer to the current standardized Dutch protocol in Table 3.

In vitro techniques have been used to elucidate the in vivo deposition patterns of INCs. As a result, researchers have used varied parameters with multiple research methods to investigate the variation in intranasal deposition. We present an overview of the conducted studies to provide insight into the knowledge of the influence of each instruction step during administration on the deposition pattern. Figure 3 illustrates an overview of the instruction steps for the administration of INC sprays and the possible variation in each instruction step.

#### Nose blowing and rinsing

To the best of our knowledge, no studies exist regarding the efficacy of nose blowing and rinsing to remove secretions prior to administration. However, several authors recommend blowing the nose because a blocked nose may diminish intranasal penetration.^30^, ^40^, ^49^, ^81^ This is also described in Dutch, English and US patient information leaflets (PILs).^82^, ^83^, ^84^, ^85^, ^86^, ^87^, ^88^, ^89^ After nose blowing, the nose can be rinsed, for example by using saline rinses. No relevant advice is given in Dutch, English and US (PILs).^82^, ^83^, ^84^, ^85^, ^86^, ^87^, ^88^, ^89^

#### Head position

The review by Benninger et al.^40^ suggests that the distribution does not vary with different head positions when using INC sprays. They therefore advise keeping the head in a neutral position.^40^ When the head is tilted back during administration, the active substance may run down the throat and causes irritation and greater systemic absorption. When the head is tilted forward, the active substance may run out of the nose. Most PILs in the Netherlands, the UK and the United States recommend tilting the head forwards.^82^, ^83^, ^84^, ^85^, ^86^, ^87^, ^88^, ^89^

#### Spray position

A Cochrane review^27^ and a meta‐analysis by Wu et al.^26^ report an increased risk of epistaxis when using an INC spray compared with a placebo or no intervention. To avoid epistaxis, the recommendation is to point the spray tip outwards, away from the nasal septum.^40^ This is also mentioned by the most Dutch, English and US PILs.^82^, ^83^, ^84^, ^85^, ^86^, ^87^, ^88^, ^89^ Another study by Benninger et al.^90^ indicates that epistaxis occurs more often on the same side as the hand that is used to spray INCs (i.e. ipsilateral hand technique). They^40^, ^90^ and Ganesh et al.^29^ therefore advise using the contralateral hand technique (i.e. right hand for the left nostril and left hand for the right nostril). Ganesh et al.^29^ showed that epistaxis was developed more often in patients who used the ipsilateral hand technique (i.e. 16 reports; 80%; *p* = .01) than in those who used the contralateral hand technique.

#### Depth of spray tip into nostril

The depth of the spray tip into the nostril during administration may influence the deposition pattern. Kimbell et al.^91^ showed that when deposition past the nasal valve is desired, the penetration improved when the nozzle was positioned 1 cm into the nostril compared with 0.5 and 1.5 cm. Most PILs in the Netherlands, the UK and the United States do not describe how deep the spray tip should be inserted into the nostril during administration. Certain PILs advise not to insert the spray tip too far into the nose, without defining precisely what is meant by “not too far.”^82^, ^83^, ^84^, ^85^, ^86^, ^87^, ^88^, ^89^

#### Closing the contralateral nostril

To the best of our knowledge, no studies have suggested that closing the other nostril while administering an INC spray affects deposition and efficacy, but most Dutch, English and US PILs advise doing so.^82^, ^83^, ^84^, ^85^, ^86^, ^87^, ^88^, ^89^ Inhalation may be more controlled with one nostril closed than if both nostrils remain open during inhalation.

#### Droplet‐size distribution

Intranasal corticosteroid sprays are likely to have a polydisperse droplet‐size distribution (DSD), in which droplets of various sizes are present. In nasal droplet deposition, three mechanisms play a major role: inertial impaction, gravitational sedimentation and Brownian diffusion. The aerodynamic diameter of droplets is a major determining factor in droplet deposition.^52^

Computational fluid dynamics (CFD) simulations were used to study the effect of variations in aerodynamic diameter and the effect on deposition. Kiaee et al.^92^ convey that when deposition in the turbinates is desirable, an aerodynamic diameter of 20–30 μm leads to maximal deposition. A comparable study by Schroeter et al.^93^ found that maximal deposition occurs in the central regions of the nose, including the turbinates, with an aerodynamic diameter of 10–11 μm. When the aerodynamic diameter increases further, the majority of droplets are deposited in the vestibule.^93^ Keeler et al.^94^ found deposition in comparable regions to be highest with aerodynamic diameters of 5–15 μm. These studies analysed the deposition of monodisperse droplets and provided information on which aerodynamic diameters help the particles to reach deeper into the nose and which are most influenced by inertial impaction. These results are fairly comparable and can be used to determine the desired DSD in case of polydisperse droplets. Research on the aerodynamic diameters that an intranasal spray bottle generates is limited. The DSD profile of INC sprays is determined by two factors: nasal airflow caused by the inhalation speed and spray velocity caused by the actuation force.

##### Nasal airflow and inhalation speed

In vitro studies investigated the effect of nasal airflow on DSD and the deposition pattern using CFD simulations. Schroeter et al.^93^ reported that deposition is influenced by droplet size in combination with airflow. A high airflow (30 L/min) leads to a peak deposition in the turbinates with aerodynamic diameters of 7–8 μm, a medium airflow (15 L/min) leads to a peak deposition in the turbinates with aerodynamic diameters of 10–11 μm, and a low airflow (7.5 L/min) leads to a peak deposition in the turbinates with aerodynamic diameters of 17–18 μm.^93^ Garlapi et al.^95^ assumed an airflow rate of 17.4 L/min as a steady‐state laminar flow. With this airflow, deposition beyond the nasal valve increased by 10–20 times compared with no airflow.^95^

The patient's inhalation speed influences the nasal airflow. In vivo studies by Tay et al.^96^ and Kimbell et al.^91^ conveyed that a gentle inspiration, similar to the steady‐state laminar flow (15 L/min) in CFD studies, leads to a better distribution beyond the nasal valve. Sniffing is not recommended because it may lead to turbulence in the nasal cavity.^93^, ^96^ Homer and Raine^97^ also suggest that inhaling vigorously does not improve deposition on the ciliated epithelium of the nasal cavity.

Remarkably, with a higher airflow, especially smaller, droplets are observed to reach deeper into the nose.^93^ This suggests that larger droplets are lost owing to the higher flow, resulting in early deposition by inertial impaction. With a lower airflow, more and larger droplets are detected deeper in the nose. The relationship between the airflow rate and DSD is thus underlined. Most PILs in the Netherlands, the UK and the United States recommend breathing in gently when activating the spray.^82^, ^83^, ^84^, ^85^, ^86^, ^87^, ^88^, ^89^

##### Spray velocity and actuation force

The spray velocity is the result of the patient's actuation force of the INCs spray device, which affects the DSD and the distribution pattern. When the spray is activated vigorously, a different plume with varying droplet aerodynamic diameters is generated, compared with a gentle activation. In the in vitro CFD study, Kiaee et al.^92^ varied spray velocity between 0 (i.e. relative to the inspiratory flow) and 20 m/s with a continuous airflow rate of 15 L/min. Maximum deposition in the turbinates was obtained with low to zero injection velocity, and deposition decreased as injection velocity increased above 5–10 m/s.^92^ When interpreting these results, it is important to consider that generation of zero injection velocity when activating the spray is highly unlikely in clinical practice. Dayal et al.^98^ found that when the mechanical actuation force increased (from 3 to 7 kg), DSD profiles with smaller droplet sizes were created. A mechanical actuation force of 4.5 kg matched best with the average hand‐actuated DSD profiles.^98^

A limitation when translating these in vitro study results into in vivo research is that there is a certain extent of mismatch between the generated plume and the anatomy of the nose. There is thus no possibility of creating a plume in the nasal cavity like that examined in the in vitro studies. Given the anatomy of the nose, and in particular the narrow passage of the valve, predicting the correct deposition based on plume geometry may be challenging.^99^

#### Exhalation

No studies were found regarding the effect of exhaling through the mouth or nose after INC spray administration on drug efficacy or drug loss. When exhaling through the nose after administration, we expect that an amount of active substance will be lost with exhalation. Most of Dutch, English and US PILs recommend exhaling through the mouth after administration.^82^, ^83^, ^84^, ^85^, ^86^, ^87^, ^88^, ^89^

#### Variation in spray devices

Although it is plausible that the length of the spray tip and nozzle configuration plays a role in the nasal distribution, research is lacking on the comparison between spray devices.^40^

### 5.7 Role of administration instructions

Aside from the importance of determining which administration technique leads to the ideal particle deposition and thereby the highest efficacy, it is important to consider which administration technique the patient knows and how the available sources describe the instructions. In scientific research, scant attention is paid to this. In recent years, the administration technique instructions in PILs, via healthcare providers and via instruction videos on YouTube have been inconsistent and of insufficient quality.^83^, ^100^, ^101^ Teaching a proper administration technique may reduce the risk of local side effects, which may lead to better treatment adherence.^7^, ^29^, ^30^, ^81^ In many countries, local initiatives have been developed to standardize correct inhalation techniques. For example, in the Netherlands, the Lung Alliance Netherlands was formed in 2009 to ensure the prevention and treatment of chronic lung diseases is controlled. Since 2019, the treatment of AR has become a focus area and a standardized protocol for INC administration has been developed based on the existing literature (Table 3).^102^

**TABLE 3:** Steps for administration of INC sprays as described in the standardized Dutch protocol.^102^

Steps for priming	
1	Shake the spray
2	Remove the dust cap
3	Place thumb under the bottle and place index and middle fingers around the nozzle
4	Point the nozzle away
5	Squirt a few sprays in the air
Steps for daily use	
6	Blow the nose
7	Shake the spray
8	Remove the dust cap
9	Place thumb under the bottle and place index and middle fingers around the nozzle
10	Keep the head straight
11	Close the other nostril
12	Point the end of the nozzle slightly outwards, away from the septum
13	Use contralateral hand position
14	Squirt a spray of mist while breathing in gently
15	Breathe out through the mouth
16	Repeat for the other nostril
17	Wipe the nozzle with a tissue
18	Replace the dust cap
19	Clean the nozzle once a week with warm water and let dry

### 5.8 Role of breath‐actuated powder inhalation devices

In the case of INC sprays, the airflow rate is determined by the inhalation speed and the spray velocity caused by the actuation force. Studies have suggested that larger droplets are lost by inertial impaction at the entrance of the nose caused by an airflow that is too high. Lower airflow rates lead to the deposition of more and larger droplets in the turbinates.^93^ In case of breath‐actuated nasal inhalers, the effect of spray velocity caused by the actuation force disappears.^103^ It is hypothesized that more and larger droplets deposit deeper into the nose due to the lower airflow rates; however, few studies have confirmed this hypothesis.

## 6 IMPLICATIONS FOR RESEARCH AND HEALTHCARE

This review describes various aspects related to INCs for the treatment of AR. Research on certain subjects is extensively available in the scientific literature, whereas limited research is available on other subjects. Table 4 presents the main findings. Table 5 summarized several knowledge gaps that can be used to set up future research.

**TABLE 4:** Main findings

Subject		Main message
AR		Global health problem that affects children and adults and influences Q when symptoms are uncontrolled
Treatment options		To control symptoms different pharmacological treatment options are available including INCs, antihistamines, leucotriene antagonists, decongestants, anticholinergics, chromones, saline rinses and immunotherapy
Efficacy of INCs	Compared to other pharmacological treatment options	INCs have been found to be the most effective therapy for moderate to severe AR symptoms
	Subjective (patient‐reported) outcome measures	INCs have proven to be effective against nasal and ocular symptoms and to improve QoL
	Objective outcome measures	PNIF, acoustic rhinometry and rhinomanometry can be used as measure for nasal obstruction. INCs show significant improvement of PNIF
	Real‐world effectiveness	The number of studies on real‐world effectiveness is limited, but overall the efficacy of INCs has been confirmed
	Regular and as‐needed therapy	INCs may be effective as as‐needed therapy for mild AR symptoms; however, regular use gives greater benefits than as‐needed therapy in total nasal symptoms score and disease‐specific QoL
Safety of INCs	Local adverse events	Local irritation and dryness of the nose and throat, and sneezing after administration are common local side effects of INCs, such as epistaxis and atrophy of the nasal mucosa or septal perforation, which are more severe and rarer adverse events
	Systemic adverse events	Adequate attention for (serious) systemic adverse events is important, including affecting the hypothalamic–pituitary–adrenal axis; affecting the growth of children; reducing bone mineral density; elevating the intraocular pressure; and developing cataract, glaucoma or chorioretinopathy
MOA		In reaction to allergic stimuli, INCs block the synthesis and release of inflammatory mediators and thereby reduce the influx of inflammatory cells into the nasal mucosa
Desired distribution pattern		No uniform conclusions regarding the desired deposition pattern of INCs could be drawn
Identified deposition pattern		Studies found different deposition patterns including: particles mainly deposit in the inferior and middle turbinates, particles mainly impinge on the non‐ciliated mucosal walls of the vestibule and the narrow valve, particles mainly deposit in the anterior non‐ciliated part of the nose and the head of the inferior turbinate and a small fraction reaches the middle turbinate
Administration technique	Steps of the administration technique	Administration steps include nose blowing, nose rinsing, head positioning, spray positioning, depth of the spray into the nostril, closing of the contralateral nostril, DSD determined by nasal airflow and spray velocity and exhalation
	The influence on the deposition pattern	No definitive conclusions could be drawn regarding how each step of the administration technique affects INCs particle deposition
Administration instructions		Instructions about the administration technique of INCs in PILs, via healthcare providers and via instruction videos on YouTube are inconsistent and of insufficient quality. Teaching a proper administration technique may reduce the risk of local side effects, which may lead to better treatment adherence
Breath‐actuated powder inhalation devices		The effect of spray velocity caused by the actuation force disappears. It is hypothesized that more and larger droplets deposit deeper into the nose due to the lower airflow rates

Abbreviations: AR, allergic rhinitis; DSD, droplet‐size distribution; INCs, intranasal corticosteroids; MOA, mode of action; PIL, patient information leaflet; PNIF, peak nasal inspiration flow; QoL, quality of life.

**TABLE 5:** Gaps in knowledge and recommendations for future research.

INCs particle deposition	Administration technique	Breath‐actuated powder inhalers
Optimise the knowledge about the most effective deposition pattern of INCs	Optimise the knowledge about the influence of the administration technique of INCs on deposition patterns and DSD profiles. Include and vary each individual instruction step: Nose blowing prior to administration Nose rinsing prior to administration Length of the spray tip Nozzle configuration Depth of the spray into the nostril during administration Closing the contralateral nostril during administration Head position during administration Inhalation during administration Actuation force of the spray during administration Exhalation after administration	Analyse the DSD profiles and deposition patterns of breath‐actuated powder inhalers of INCs due to the absence of spray velocity during administration
Analyse which DSD profile of INCs leads to the most effective deposition pattern		

Abbreviations: DSD, droplet‐size distribution; INCs, intranasal corticosteroids.

## 7 CONCLUSION

This review provides an overview of the available literature on INCs for the treatment of AR. Guidelines describe the pharmacological treatment options for AR and the application of these treatments for mild or moderate‐to‐severe symptoms. INCs are considered to be the most effective therapy for moderate to severe AR symptoms. Subjective (i.e. patient‐reported) outcome measures are used to determine the efficacy of INCs; however, objective outcome measures are now rarely used. Safety is extensively investigated in the literature and INCs are generally well‐tolerated; however, adequate attention for serious (systemic) adverse events is important in daily clinical practice. Although INCs are considered as effective and safe, their efficacy and safety may also depend on nasal particle deposition, which the administration technique affects. However, little attention is paid to this in scientific literature. Future research is necessary, and extensive research on the effect of the administration technique will provide insight into the most effective and safe deposition pattern. In combination with adequate attention for providing instructions for proper administration, this should result in better use, the occurrence of fewer adverse events and improved care for patients with AR.

## AUTHOR CONTRIBUTIONS

The original manuscript idea was set up by all authors. The literature search was carried out by CR; hereby ENR and TWV fulfilled an advisory and supervisory role. The design of the manuscript was set up by all authors and CR elaborated the design into this manuscript. All authors contributed to drafting and revising the manuscript; they gave the final approval of the version to be published and agree to be accountable for all aspects of the work.

## CONFLICT OF INTEREST

The authors declare no relevant conflicts of interest.

## Data Availability

Data sharing not applicable to this article as no datasets were generated or analysed during the current study.
